# Simultaneous Bilateral Pediatric Nephrectomies: Indications, Approach and Outcomes Over a 15‐Year Period

**DOI:** 10.1111/petr.70072

**Published:** 2025-03-31

**Authors:** A. M. Lombardo, M. Stout, A. Zann, D. McLeod, S. Alpert, V. R. Jayanthi, D. DaJusta, C. B. Ching

**Affiliations:** ^1^ Department of Urology The Ohio State University Wexner Medical Center Columbus Ohio USA; ^2^ Department of Urology Monroe Carell Jr. Children's Hospital, Vanderbilt University Medical Center Nashville Tennessee USA; ^3^ Department of Pediatric Urology Kidney and Urinary Tract Center, Nationwide Children's Hospital Columbus Ohio USA; ^4^ Section of Pediatric Urology Children's Hospital and Medical Center, University of Nebraska Medical Center Omaha Nebraska USA

**Keywords:** bilateral nephrectomy, nephrectomy, pediatric urology, renal transplant, robotic

## Abstract

**Background:**

Reports on bilateral simultaneous native nephrectomies in the pediatric population are lacking. This study evaluates indications and outcomes of a tertiary care pediatric hospital's single center experience with bilateral simultaneous native nephrectomies over 15 years.

**Methods:**

A retrospective chart review of pediatric patients (< 21 years old) who underwent bilateral simultaneous native nephrectomy from January 1, 2009, to August 1, 2024, at a single institution was performed for surgical indications, approach, and outcomes.

**Results:**

Ten patients were identified. Mean age at bilateral simultaneous nephrectomy was 9.6 years (range 14 months–19 years). Surgical indications included hypertensive crisis in four, nephrotic syndrome in three, Polycythemia Vera in one, recurrent urinary tract infections (UTI) in one, and significant hydroureteronephrosis in one patients. Prior to nephrectomy, six patients were on hemodialysis, two patients were on peritoneal dialysis (PD), and two patients were not on any form of dialysis. Of the 10 patients, four underwent surgery during an inpatient admission for an acute exacerbation of the listed indication. Three cases were done robotically (transperitoneal), and seven were performed open. Both patients on PD preoperatively underwent open retroperitoneal surgery and were able to restart PD on postoperative day 1. There was one complication in a patient with recurrent UTIs who developed an intra‐abdominal abscess, requiring percutaneous drainage.

**Conclusions:**

Bilateral simultaneous native nephrectomy is a safe and effective way to manage conditions associated with end‐stage renal disease in pediatric patients. In our experience, this can be done by an open or minimally invasive approach.

## Graphical abstract

Our single‐institution 15‐year experience with simultaneous bilateral native nephrectomy (BNN) in pediatric patients demonstrates utility and safety. We discuss considerations of BNN relative to timing of transplantation, an area of conversation. We demonstrate that a minimally invasive approach is feasible, and the surgical approach should consider the route of current or planned dialysis.

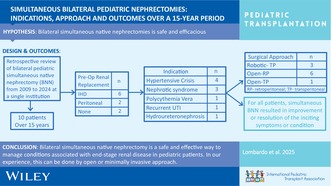

## 1 Introduction

Patients may require bilateral nephrectomy for a variety of reasons. The etiology of pathology may be related to primary medical renal disease or to structural abnormalities of the kidneys themselves. Indications differ slightly between adults and pediatric patients related to the differences in pathology encountered in each population, such as polycystic kidney disease in adults versus tubular and glomerular abnormalities leading to polyuria and proteinuria as well as congenital anatomic kidney and urinary tract abnormalities one may see in children [1, 2, 3, 4, 5].

There is some controversy regarding BNN in the pediatric population, perhaps resulting in—and from—the relative infrequency in which it is performed. Benefits of BNN for the treatment of disease states have been documented in case reports describing outcomes after BNN in primary intrinsic kidney diseases, including oxaluria, polyuria, and nephrotic syndromes [6, 7, 8]. Though the timing of BNN relative to transplant is a topic of discussion, evidence suggests that pretransplant BNN can offer a benefit to graft survival and transplant blood flow [5, 9]. In addition, BNN performed for the indication of certain disease states may improve surgical fitness for later graft implantation. It has been hypothesized that BNN prior to transplant could be associated with subsequent anemia resulting in the need for blood transfusions, risking sensitization which could complicate transplant [10]. In addition, there can be concern with removing potential sources of volume management and blood pressure control if the native kidneys are still making some urine and providing a source for the renin‐angiotensin‐aldosterone system [5, 11]. The timing of nephrectomy relative to transplantation is an area of conversation, with further consideration regarding staged versus synchronous bilateral nephrectomy.

To aid in decision‐making, one looks to the literature, for which there are few publications. Most of the studies that describe indications and outcomes of pediatric bilateral nephrectomy do so with simultaneous BNN as a subgroup of a larger cohort [3, 4, 5]. The goal of this study was to specifically review the experience over a 15‐year time period of simultaneous BNN at a tertiary U.S. pediatric hospital.

## 2 Patients and Methods

This study was conducted by retrospective chart review of pediatric patients who underwent simultaneous BNN from January 1, 2009, to August 1, 2024, at a single tertiary care children's hospital. Patients were identified using CPT codes 50 545 (radical nephrectomy) and 50 548 (nephrectomy with partial ureterectomy) and ICD‐9‐CM code 55.54 (bilateral nephrectomy) and ICD‐10‐PCS codes 0TT20ZZ (open resection of bilateral kidneys) and TT24ZZ (percutaneous endoscopic approach resection of bilateral kidneys). Output from CPT‐generated cases was cross‐checked with the internal department‐generated weekly operating room schedule and was only included if there was a simultaneous bilateral surgical procedure. Patients with solitary kidney or staged bilateral nephrectomy were excluded. Patient demographic data and relevant clinical details, including operative reports, were retrospectively reviewed and extracted from the electronic medical record. Clinical data included patient age, medical comorbidities, indications for surgery, dialysis and transplant status, operative details, and intraoperative and postoperative complications. Statistical analysis of descriptive outcomes was performed using Microsoft excel. A comparison of operative time, blood loss, and length of postoperative hospital stay between robotic and open surgical approaches using *t*‐test or Mann–Whitney *U*‐test was performed when appropriate based on normative values with GraphPad software. A *p* value of < 0.05 was considered significant. This study was approved by the Institutional Review Board (IRB16‐00249).

## 3 Results

Ten patients who underwent simultaneous BNN were identified. Surgery was performed by five different attending urologists. The mean age of the patients at the time of nephrectomy was 9.6 years, ranging from 14 months to 19 years. The indications for surgery and preoperative dialysis status are outlined in Table 1. Of the 10 patients, four underwent surgery during an inpatient admission for an acute exacerbation (two for hypertensive crisis and two for nephrotic syndrome). Three cases were done robotically (all transperitoneal), and seven were performed open, six of which were performed retroperitoneal, and one of which was performed transperitoneal. Both patients on PD preoperatively underwent open retroperitoneal surgery and were able to resume peritoneal dialysis (PD) the evening of postoperative day 1. One patient had a PD catheter placed at the time of open retroperitoneal BNN. Of the patients undergoing transperitoneal surgery, one patient with Polycythemia Vera had already undergone transplantation, one started IHD after surgery until living related transplant 3 months later, and two were already on IHD.

**TABLE 1:** Characteristics of patients undergoing bilateral simultaneous nephrectomies.

Characteristic	Prevalence
Indication for bilateral nephrectomy	
Hypertensive crisis	4
Nephrotic syndrome	3
Polycythemia vera	1
Recurrent UTI	1
Significant hydroureteronephrosis	1
Dialysis status prior to nephrectomy	
Intermittent hemodialysis	6
Peritoneal	2
None	2
Surgical approach	
Robotic	3
Transperitoneal	3
Open	7
Retroperitoneal	6
Transperitoneal	1
Transplantation status	
Prior to surgery	1
Post nephrectomy	8

Operative outcomes and postoperative length of stay are summarized in Table 2. One patient had a protracted postoperative course resulting in a postoperative stay of 104 days. He was a 14‐month‐old child who had been hospitalized for complications of his nephrotic syndrome initially, leading to his acute requirement for BNN. Postoperatively, he became ill with adenovirus infection, a deep vein thrombosis, and a pulmonary embolism. His protracted course was felt to be a result of his severe nephrotic syndrome versus being directly a result of his surgery. There was one postoperative complication specifically due to the BNN in a patient with recurrent UTIs who developed an intra‐abdominal abscess requiring percutaneous drainage. Of note, this patient had complex anatomy, including a remote history of left‐to‐right transureteroureterostomy with a single reimplanted right ureter into the bladder by an outside provider. It was unknown whether this patient had urinary reflux into that right ureter as he had been managed with chronic indwelling stents for concern of chronic obstruction and did not have a voiding cystourethrogram (VCUG) in our system at the time he had transferred care to our hospital. At the time of simultaneous BNN, the single ureteral distal stump was oversewn. The patient who underwent PD catheter placement at the time of open retroperitoneal BNN developed *Staphylococcus aureus* peritonitis on postoperative day 28, which was not clearly related to the BNN and was managed with intravenous antibiotics. For all 10 patients, simultaneous BNN resulted in improvement or resolution of the inciting symptoms or condition. Specifically, the patient with preoperative UTIs has been infection‐free since BNN; the patient with PV has had normalization of his erythrocytosis; and of the four patients who had BNN for hypertensive crises, two were weaned off of all antihypertensive medications by 8 months postoperatively, one was able to decrease his total antihypertensives from four to three, and one who was requiring intravenous antihypertensive drip at surgery was able to be transitioned to two antihypertensive oral medications. Eight patients have since been transplanted; one was transplanted prior to surgery; one patient passed away before transplantation.

**TABLE 2:** Comparison of metrics between open vs. robotic approach for bilateral simultaneous nephrectomies.

Outcome	Open (*n* = 7)	Robotic (*n* = 3)	*p*
Median operative time in minutes (IQR)	259 (188–393)	513 (317–524)	0.0531
Median blood loss in milliliter (IQR)	100 (100–138)	50 (10–383)	0.6286
Median length of postoperative stay in days (IQR)	8 (5–21)	4 (3–19)	0.2917

## 4 Discussion

The data support better long‐term patient survival for kidney transplant recipients compared with those who remain on dialysis [1]. Since the first successful kidney transplantation in 1954, there have fortunately been advances regarding immunology, allowing more donors and patients to be treated. Unfortunately, the incidence of end‐stage renal disease (ESRD) in the adult American population has continued to rise with the most common etiology of ESRD/chronic kidney disease being hypertension and diabetes followed by autosomal dominant polycystic kidney disease (ADPKD) [1, 2]. Pediatric renal transplantation is different from adult transplantation in a variety of factors, including the etiology of CKD/ESRD. A large percentage of children with CKD develop the disease early in life, with congenital renal disorders, such as obstructive uropathy, aplasia, hypoplasia, or dysplasia compromising almost half of all cases [2, 12].

A patient may require BNN due to a variety of indications. In adult patients, these may include primary renal malignancy or metastasis to the kidney [13, 14, 15], polycystic kidney disease [16, 17], uncontrolled hypertension [18], and, less commonly, erythrocytosis [19, 20]. In some with known end‐stage renal disease, BNN can decrease renal allograft rejection [9, 21]. In the pediatric population, indications for BNN can include primary hyperoxaluria [22], significant polyuria [5, 7], uncontrolled hypertension [3, 4], congenital anomalies resulting in recurrent UTI or sepsis [3, 5] and nephrotic syndromes, including focal segmental glomerular sclerosis (FSGS) [2, 5, 23].

Within the kidney transplant literature, timing of BNN relative to transplant (pre‐, concurrently, or post‐transplant) is debated. When nephrectomy is completed prior to transplantation, there may be renal graft complications related to fluid volume management and time spent on dialysis, especially if dialysis was not needed prior to native nephrectomy. On the contrary, in patients who receive nephrectomy after transplantation, immunosuppression can contribute to perioperative complications [24, 25]. Yet some studies suggest that pretransplant BNN may have a positive outcome on transplantation. In the adult literature, Sanfilippo et al. [21] report increased overall graft survival associated with pretransplant BNN in a prospective study of 2808 first‐time renal allograft recipients compared with transplant recipients who had concurrent BNN at time of transplant and those not undergoing BNN at all, independent of the etiology of renal failure or indication for nephrectomy (*p* < 0.003). The improved graft survival seemed immune mediated given there was specifically a lower incidence of acute transplant rejection within the pretransplant BNN cohort compared with the other cohorts (*p* < 0.007) [21]. In addition, pretransplant adult BNN has been proclaimed beneficial in specific disease states, such as autosomal dominant polycystic kidney disease where performing nephrectomies may be necessary to increase intra‐abdominal working space for transplantation [26, 27].

BNN with simultaneous kidney transplant can be a viable option, but can incur higher surgical risk associated with longer operative time and subsequent anesthetic exposure and longer length of hospital stay compared with staged BNN/transplantation [28, 29]. In high‐volume centers, BNN with simultaneous kidney transplantation can offer the benefit of singular hospital stay and recovery period [28, 29]. In one of the largest cohorts of patients with Autosomal Dominant Polycystic Kidney Disease and renal transplantation (*n* = 594) at a single center, three groups were analyzed: renal transplant‐only, simultaneous BNN and transplant, and pretransplant bilateral BNN [30]. Importantly, graft survival at 10 years follow up was not significantly different among groups (*p* = 0.86). More wound complications were seen in pretransplant BNN (25.9% vs. 11.1% in transplant alone, 5.1% simultaneous BNN and transplant; *p* = 0.03). Simultaneous BNN patients had a higher incidence of renal vascular thromboses (4.4% vs. 1.3% transplant alone, and 0% pretransplant BNN; *p* = 0.04). Interestingly, 16.3% of the “transplant alone” cohort required nephrectomy at 10 years [30]. Based on this study, one may conclude that the decision for pretransplant BNN versus simultaneous BNN should account for individual patient risk factors and the availability of living donor which logistically may lend to pre‐planned simultaneous BNN with transplant. Yet, again, the majority of the research has been done in adult patients with little information on performance in the pediatric population.

Another important consideration in BNN is whether the case should be completed by transperitoneal or retroperitoneal approach, as this may be influenced by the presence of active or planned PD. Avoiding a transperitoneal approach may be desirable in the setting of PD to avoid violation of the intra‐abdominal space. The mode of dialysis did influence surgical approach in our study. Patients on PD underwent retroperitoneal surgery, resulting in limited interruption of their pre‐BNN dialysis method, with all patients on PD able to resume PD without requiring IHD postoperatively. For those on IHD, they all resumed IHD on postoperative day 1. There is some guidance in the pediatric literature regarding suggested approach given the prevalence of PD in children. Gundeti et al. [31] describe indications, technique, and outcomes of 20 patients with pediatrics who underwent laparoscopic approach for bilateral synchronous posterior prone retroperitoneoscopic nephrectomy (BSPPRN) for end‐stage renal disease (ESRD) of varying etiology. Of 20 patients, a PD catheter was concurrently placed in eight children and 10 additional children were already on PD. Their technique included two or three ports. BSPPRN was successful in 19 patients. One patient, a 6‐kg infant with large kidneys due to congenital nephrotic syndrome, had an intraoperative peritoneal tear and required conversion to an open exploration [31]. No patients required blood transfusions and there were no postoperative complications. Fifteen patients initiated PD within 24 h postoperatively. It is notable that 30% of our cases were performed robotically. Prior studies describing outcomes of minimally invasive approaches via laparoscopy or robot‐assisted technology in pediatric BNN have been sparse. Marietti et al. [32] describe four cases of laparoscopic transperitoneal single‐incision pediatric bilateral nephrectomy for various pretransplant etiologies using the Covidien SILS port. Use of a minimally invasive approach can be used successfully in the pediatric population from prior reports [25, 31, 32] and is confirmed in our experience. We did not find a difference in operative variables like operative time or blood loss between open and robotic surgical approach, nor a difference in postoperative length of stay; our statistical analysis, however, was limited by the small sample size. Larger study samples are needed for robust comparison of outcomes with respect to open techniques and approach selection is often patient‐ or surgeon‐specific.

Other limitations of our study include the retrospective nature of the design and reliance on electronic medical record documentation. Despite this, we think our study is valuable to add another series of patients with pediatrics undergoing this uncommon operation and the considerations that might guide others in performing this operation.

## 5 Conclusion

Our experience with simultaneous BNN demonstrates its utility and safety in the pediatric population, even when performed due to an acute exacerbation of the indication for BNN. We demonstrate that a minimally invasive approach is feasible and that the approach should take into consideration the form of dialysis or planned dialysis.

## Data Availability

The data that support the findings of this study are available upon request from the corresponding author. The data are not publicly available due to privacy or ethical restrictions.

## References

[1] S. Abramyan and M. Hanlon , “Kidney Transplantation,” in StatPearls [Internet] (StatPearls Publishing, 2023), https://www.ncbi.nlm.nih.gov/books/NBK567755/. 33620832

[2] M. H. Cho , “Pediatric Kidney Transplantation Is Different From Adult Kidney Transplantation,” Korean Journal of Pediatrics 61, no. 7 (2018): 205–209, 10.3345/kjp.2018.61.7.205. 30032586 PMC6106688

[3] N. Fraser , P. C. Lyon , A. R. Williams , M. T. Christian , and M. U. Shenoy , “Native Nephrectomy in Pediatric Transplantation – Less Is More!,” Journal of Pediatric Urology 9, no. 1 (2013): 84–89, 10.1016/j.jpurol.2011.12.008. 22227459

[4] A. L. Brubaker , D. J. Stoltz , A. Chaudhuri , et al., “Superior Hypertension Management in Pediatric Kidney Transplant Patients After Native Nephrectomy,” Transplantation 102, no. 7 (2018): 1172–1178, 10.1097/TP.0000000000002093. 29953422 PMC7228635

[5] F. Ghane Sharbaf , M. Bitzan , K. M. Szymanski , et al., “Native Nephrectomy Prior to Pediatric Kidney Transplantation: Biological and Clinical Aspects,” Pediatric Nephrology 27, no. 7 (2012): 1179–1188, 10.1007/s00467-012-2115-y. 22366876 PMC3362721

[6] A. Chaudhuri , O. Salvatierra , S. R. Alexander , and M. M. Sarwal , “Option of Pre‐Emptive Nephrectomy and Renal Transplantation for Bartter's Syndrome,” Pediatric Transplantation 10, no. 2 (2006): 266–270, 10.1111/j.1399-3046.2005.00435.x. 16573620

[7] D. Kravarusic , D. L. Sigalet , L. A. Hamiwka , J. P. Midgley , A. W. Wade , and S. Grisaru , “Persistent Post‐Transplant Polyuria Managed by Bilateral Native‐Kidney Laparoscopic Nephrectomy,” Pediatric Nephrology 21, no. 6 (2006): 880–882, 10.1007/s00467-006-0085-7. 16703380

[8] V. Villani , N. Gupta , N. Elias , et al., “Bilateral Native Nephrectomy Reduces Systemic Oxalate Level After Combined Liver‐Kidney Transplant: A Case Report,” Pediatric Transplantation 21, no. 3 (2017): e12901, 10.1111/petr.12901. 28261895

[9] T. M. Coffman , F. P. Sanfilippo , P. C. Brazy , W. E. Yarger , and P. E. Klotman , “Bilateral Native Nephrectomy Improves Renal Isograft Function in Rats,” Kidney International 30, no. 1 (1986): 20–26, 10.1038/ki.1986.145. 3528617

[10] C. R. Darby , D. Cranston , A. E. Raine , and P. J. Morris , “Bilateral Nephrectomy Before Transplantation: Indications, Surgical Approach, Morbidity and Mortality,” British Journal of Surgery 78, no. 3 (1991): 305–307, 10.1002/bjs.1800780313. 2021844

[11] R. E. Hofer , T. M. Kor , M. Prieto , and J. Y. Findlay , “The Perioperative Management of Simultaneous Bilateral Nephrectomy With Renal Transplantation: A Case Series,” Canadian Journal of Anaesthesia 68, no. 8 (2021): 1254–1259, 10.1007/s12630-021-01989-1. 33846909 PMC8041388

[12] B. Saeed , “Pediatric Renal Transplantation,” International Journal of Organ Transplantation Medicine 3, no. 2 (2012): 62–73. 25013625 PMC4089282

[13] C. Ketsuwan , P. Sangkum , P. Sirisreetreerux , W. Viseshsindh , S. Patcharatrakul , and W. Kongcharoensombat , “Laparoscopic Bilateral Nephro‐Ureterectomy Approach for Complete Urinary Tract Extirpation for the Treatment of Multifocal Urothelial Carcinoma in a Kidney Transplant Patient: A Case Report and Literature Review,” Transplantation Proceedings 47, no. 7 (2015): 2265–2269, 10.1016/j.transproceed.2015.07.020. 26361696

[14] T. Klatte and M. Marberger , “Renal Cell Carcinoma of Native Kidneys in Renal Transplant Patients,” Current Opinion in Urology 21, no. 5 (2011): 376–379, 10.1097/MOU.0b013e32834962bf. 21730855

[15] A. Noce , G. Iaria , O. Durante , et al., “Bilateral Native Kidney Neoplasia Detected by Ultrasound in Functionning Renal Allograft Recipient,” Archivio Italiano di Urologia, Andrologia 84, no. 4 (2012): 253–255. 23427757

[16] T. Misumi , K. Ide , T. Onoe , et al., “Incidental Renal Cell Carcinoma Presenting in a Renal Transplant Recipient With Autosomal Dominant Polycystic Kidney Disease: A Case Report,” Journal of Medical Case Reports 6 (2012): 154, 10.1186/1752-1947-6-154. 22691223 PMC3423017

[17] D. T. Glassman , L. Nipkow , S. T. Bartlett , and S. C. Jacobs , “Bilateral Nephrectomy With Concomitant Renal Graft Transplantation for Autosomal Dominant Polycystic Kidney Disease,” Journal of Urology 164, no. 3 Pt 1 (2000): 661–664, 10.1097/00005392-200009010-00011. 10953121

[18] M. J. Lerman , S. Hinton , and R. Aronoff , “Bilateral Native Nephrectomy for Refractory Hypertension in Kidney Transplant and Kidney Pancreas Transplant Patients,” International Journal of Surgery Case Reports 15 (2015): 127–129, 10.1016/j.ijscr.2015.08.001. 26348394 PMC4601950

[19] M. A. Castaneda , P. J. Garvin , J. E. Codd , and K. Carney , “Selective Posttransplantation Bilateral Native Nephrectomy. Indications and Results,” Archives of Surgery 118, no. 10 (1983): 1194–1196, 10.1001/archsurg.1983.01390100064016. 6351807

[20] F. J. Dagher , E. Ramos , A. Erslev , S. Karmi , and S. V. Alongi , “Erythrocytosis After Renal Allotransplantation: Treatment by Removal of the Native Kidneys,” Southern Medical Journal 73, no. 7 (1980): 940–942. 6992287

[21] F. Sanfilippo , W. K. Vaughn , and E. K. Spees , “The Association of Pretransplant Native Nephrectomy With Decreased Renal Allograft Rejection,” Transplantation 37, no. 3 (1984): 256–260, 10.1097/00007890-198403000-00008. 6367165

[22] E. Lee , G. Ramos‐Gonzalez , N. Rodig , S. Elisofon , K. Vakili , and H. B. Kim , “Bilateral Native Nephrectomy to Reduce Oxalate Stores in Children at the Time of Combined Liver‐Kidney Transplantation for Primary Hyperoxaluria Type 1,” Pediatric Nephrology 33, no. 5 (2018): 881–887, 10.1007/s00467-017-3855-5. 29243158

[23] G. M. C. Fuentes , C. G. Meseguer , A. P. Carrion , et al., “Long‐Term Outcome of Focal Segmental Glomerulosclerosis After Pediatric Renal Transplantation,” Pediatric Nephrology 25, no. 3 (2010): 529–534, 10.1007/s00467-009-1361-0. 19956977

[24] J. R. Kaplan , R. S. Sung , and J. S. Wolf , “Bilateral Native Nephrectomy: Before or After Renal Transplantation?,” UIJ 02, no. 01 (2008), 10.3834/uij.1939-4810.2008.12.04.

[25] S. Durham , N. Cheng , N. G. Jain , M. Stifelman , and R. Schlussel , “Bilateral Robotic Nephrectomy in a Pediatric Female Patient: A Case Report,” JU Open Plus 1, no. 5 (2023): e00019, 10.1097/JU9.0000000000000021.

[26] M. D. Tyson , E. S. Wisenbaugh , P. E. Andrews , E. P. Castle , and M. R. Humphreys , “Simultaneous Kidney Transplantation and Bilateral Native Nephrectomy for Polycystic Kidney Disease,” Journal of Urology 190, no. 6 (2013): 2170–2174, 10.1016/j.juro.2013.05.057. 23727414

[27] A. Dinckan , H. Kocak , A. Tekin , and S. Yucel , “Concurrent Unilateral or Bilateral Native Nephrectomy in Kidney Transplant Recipients,” Annals of Transplantation 18 (2013): 697–704, 10.12659/AOT.889377. 24356642

[28] A. Kramer , J. Sausville , A. Haririan , S. Bartlett , M. Cooper , and M. Phelan , “Simultaneous Bilateral Native Nephrectomy and Living Donor Renal Transplantation Are Successful for Polycystic Kidney Disease: The University of Maryland Experience,” Journal of Urology 181, no. 2 (2009): 724–728, 10.1016/j.juro.2008.10.008. 19091353

[29] A. D. Martin , K. L. Mekeel , E. P. Castle , et al., “Laparoscopic Bilateral Native Nephrectomies With Simultaneous Kidney Transplantation,” BJU International 110, no. 11c (2012): E1003–E1007, 10.1111/j.1464-410X.2012.11379.x. 22882539

[30] E. I. Grodstein , N. Baggett , S. Wayne , et al., “An Evaluation of the Safety and Efficacy of Simultaneous Bilateral Nephrectomy and Renal Transplantation for Polycystic Kidney Disease: A 20‐Year Experience,” Transplantation 101, no. 11 (2017): 2774–2779, 10.1097/TP.0000000000001779. 29064957

[31] M. S. Gundeti , A. Taghizaedh , and I. Mushtaq , “Bilateral Synchronous Posterior Prone Retroperitoneoscopic Nephrectomy With Simultaneous Peritoneal Dialysis: A New Management for End‐Stage Renal Disease in Children,” BJU International 99, no. 4 (2007): 904–906, 10.1111/j.1464-410X.2006.06700.x. 17233807

[32] S. Marietti , N. Holmes , and G. Chiang , “Laparoendoscopic Single‐Site (LESS) Bilateral Nephrectomy in the Pretransplant Pediatric Population,” Pediatric Transplantation 15, no. 4 (2011): 396–399, 10.1111/j.1399-3046.2011.01504.x. 21585628

